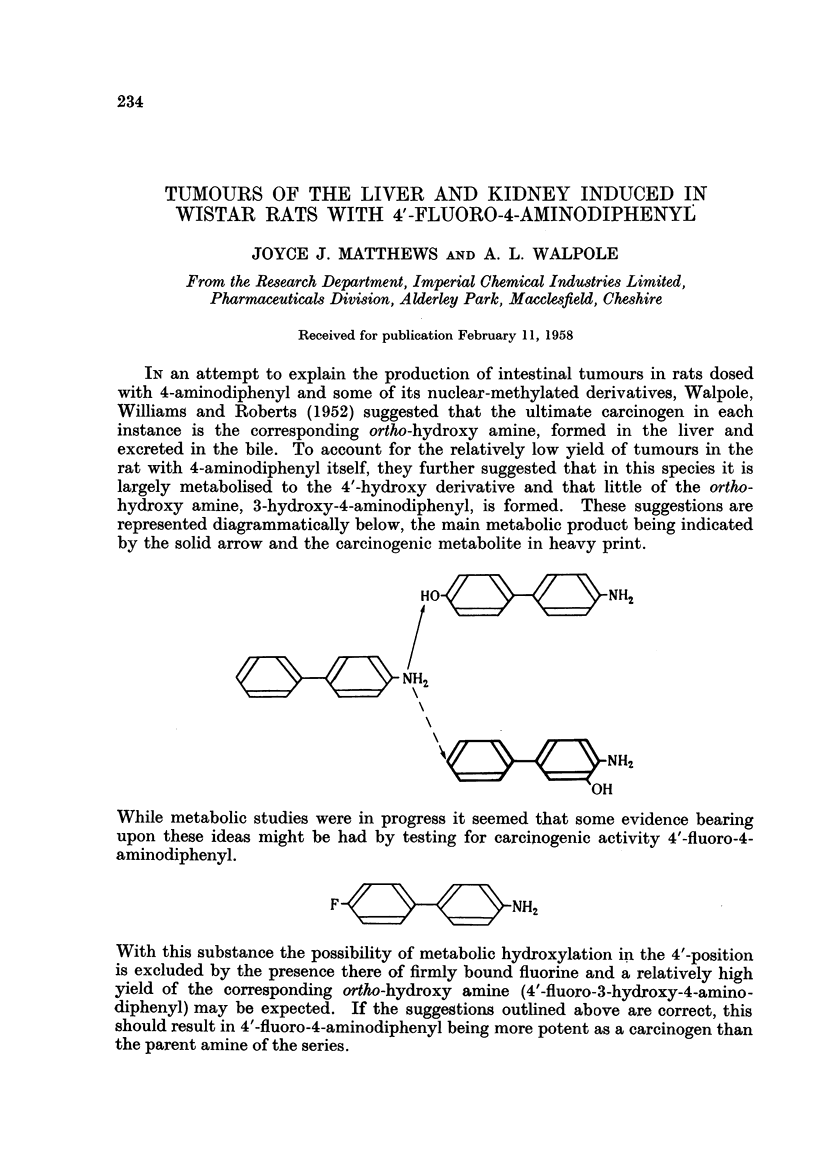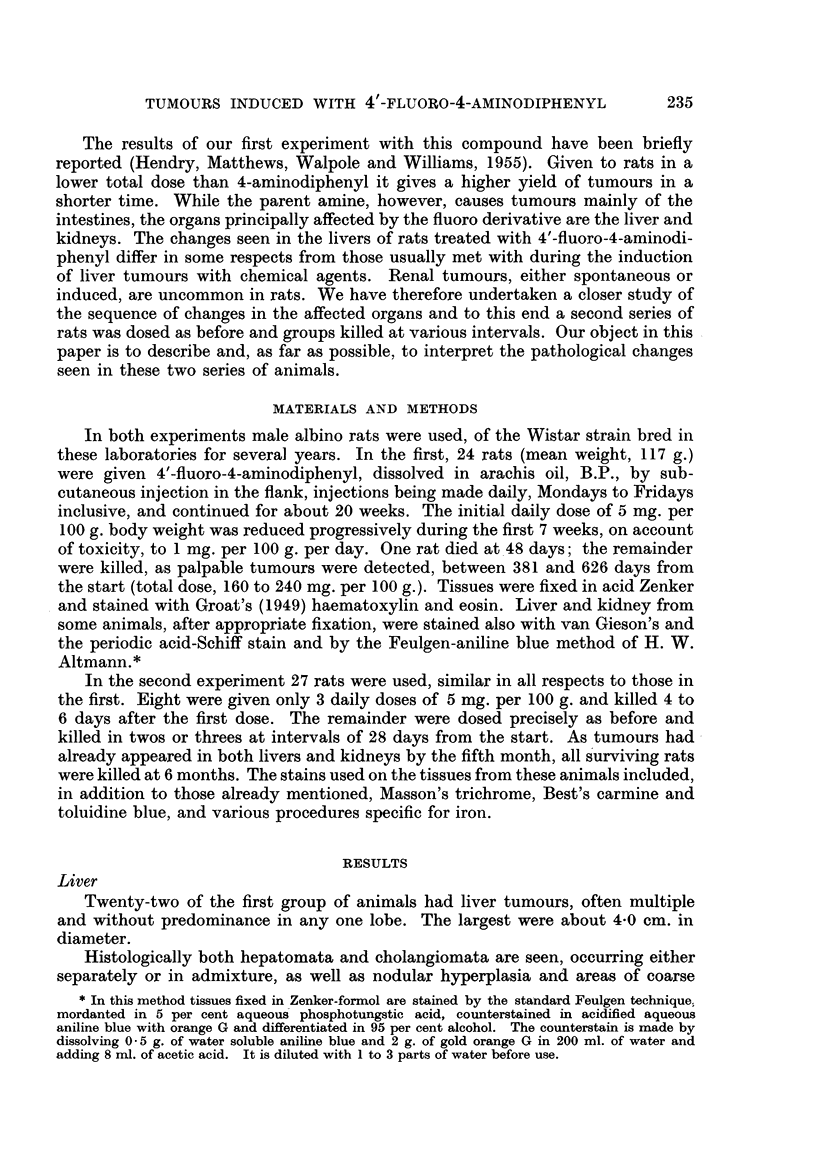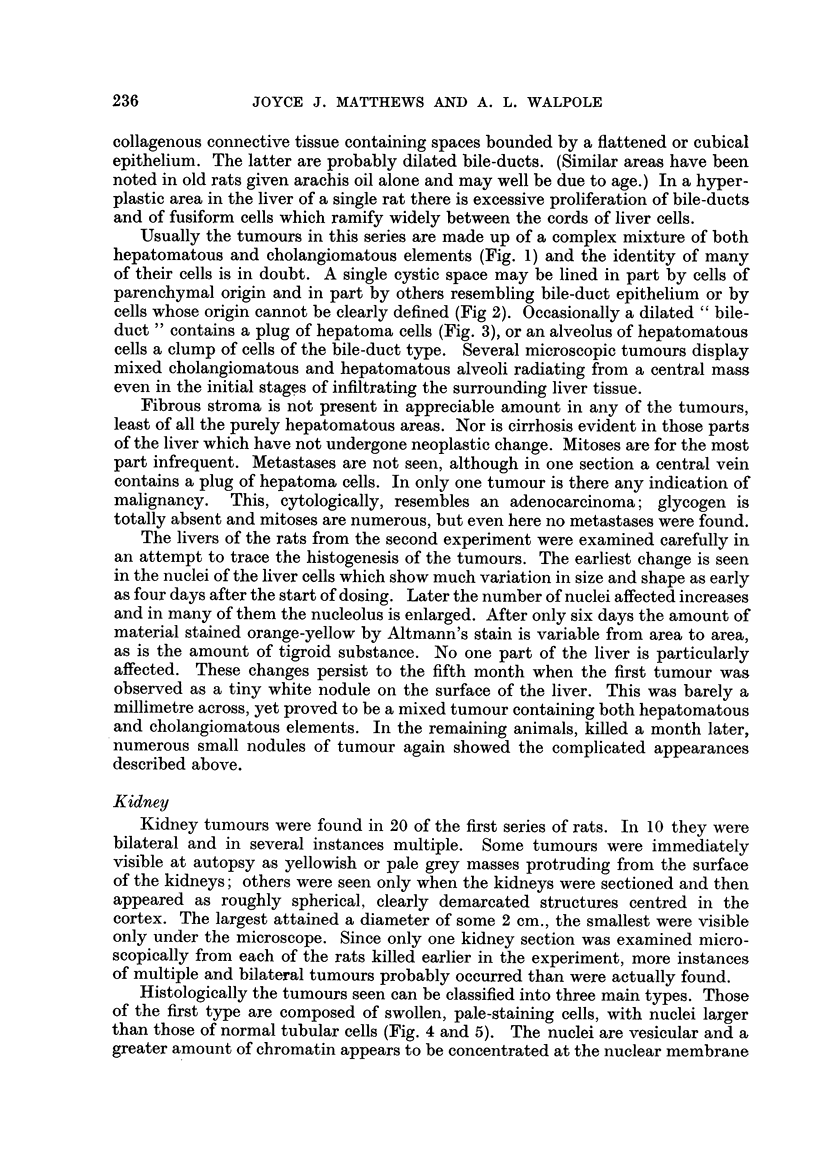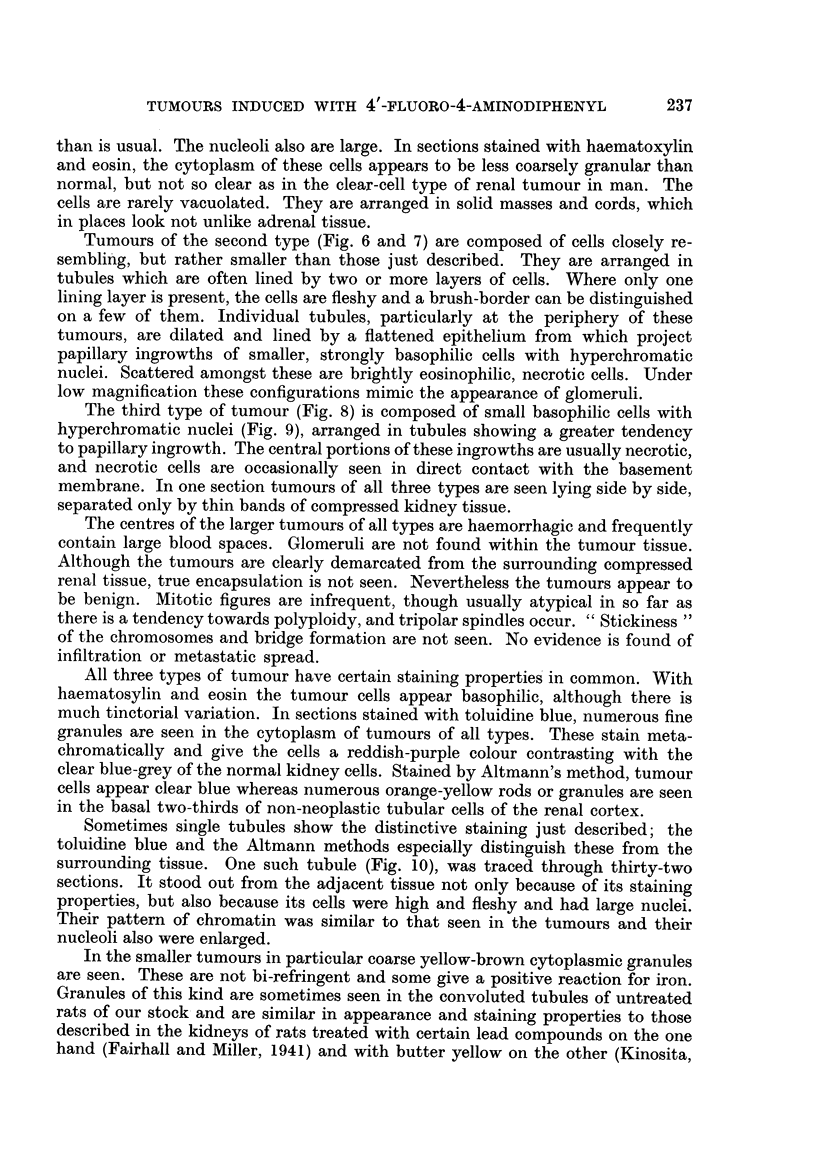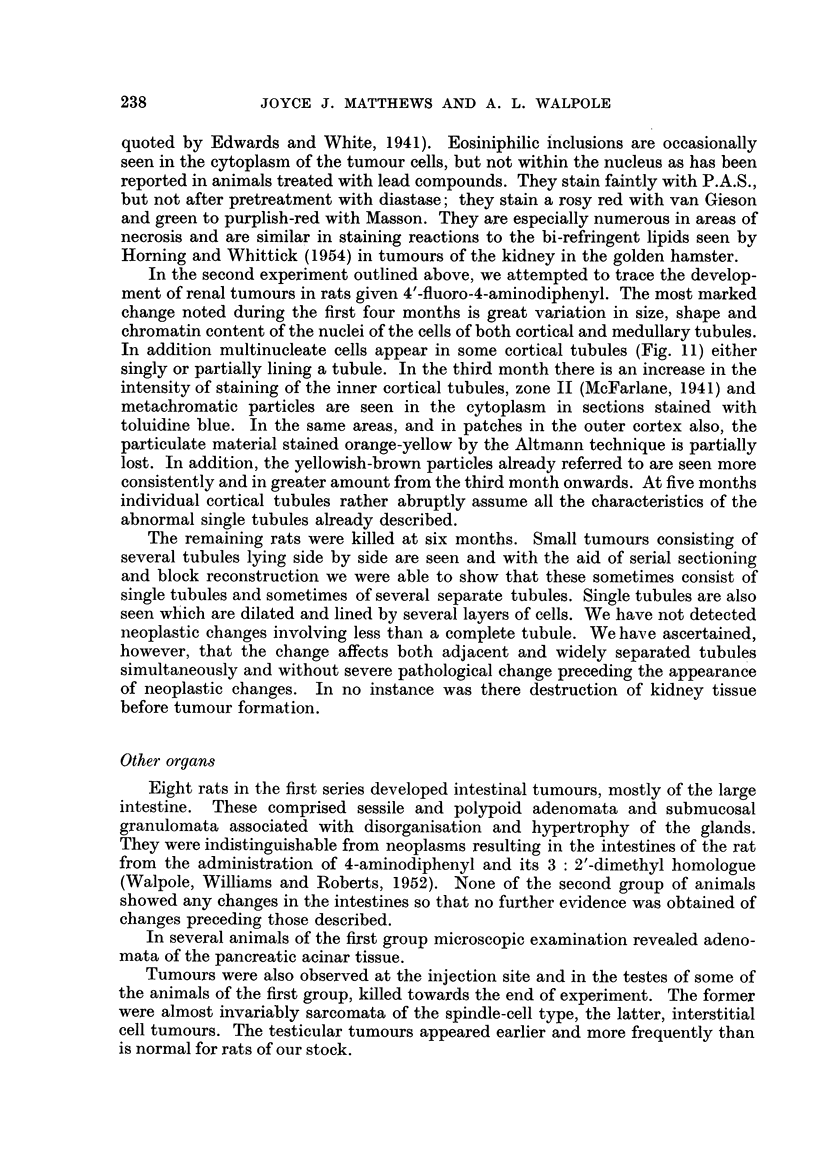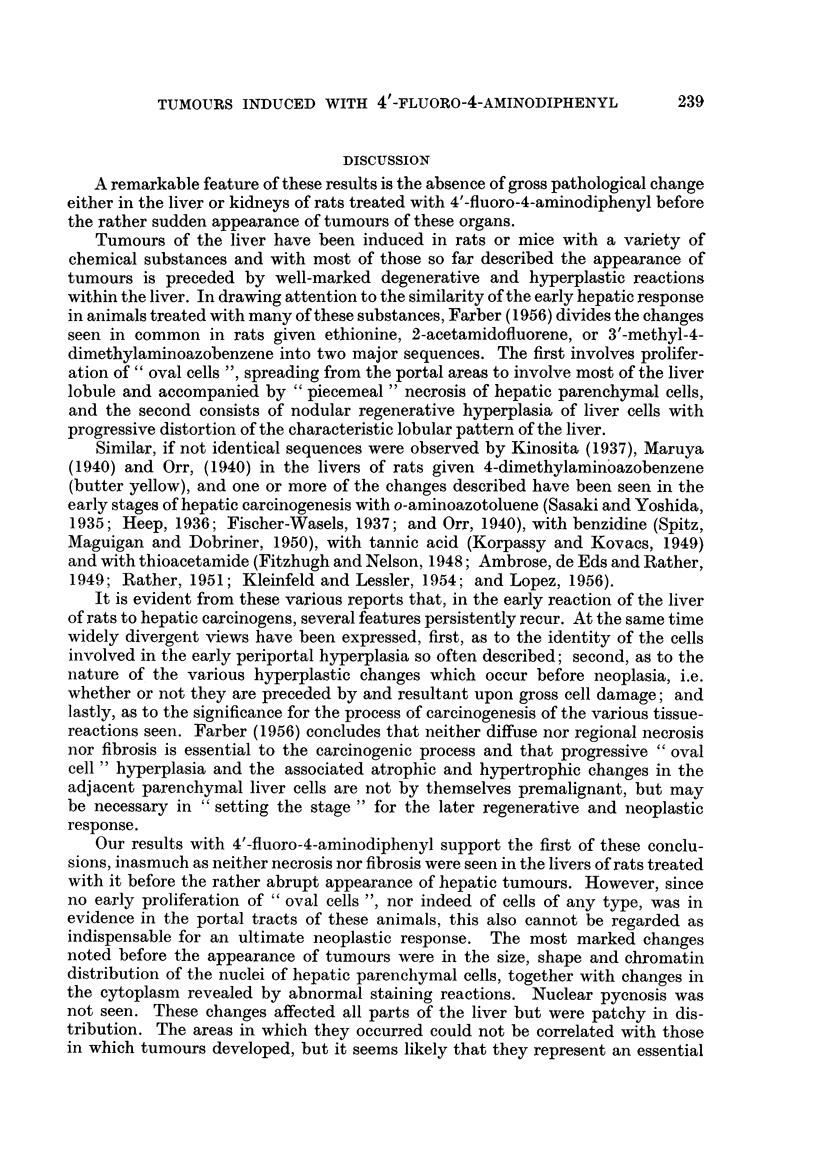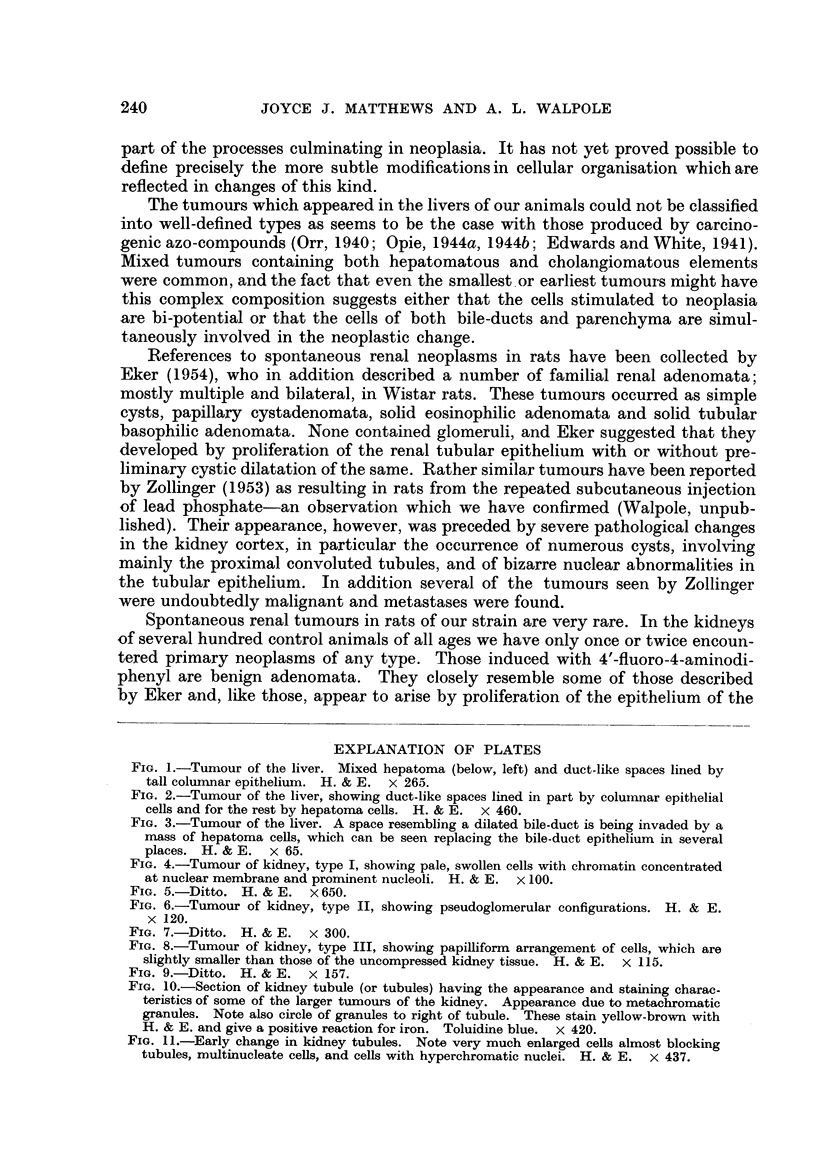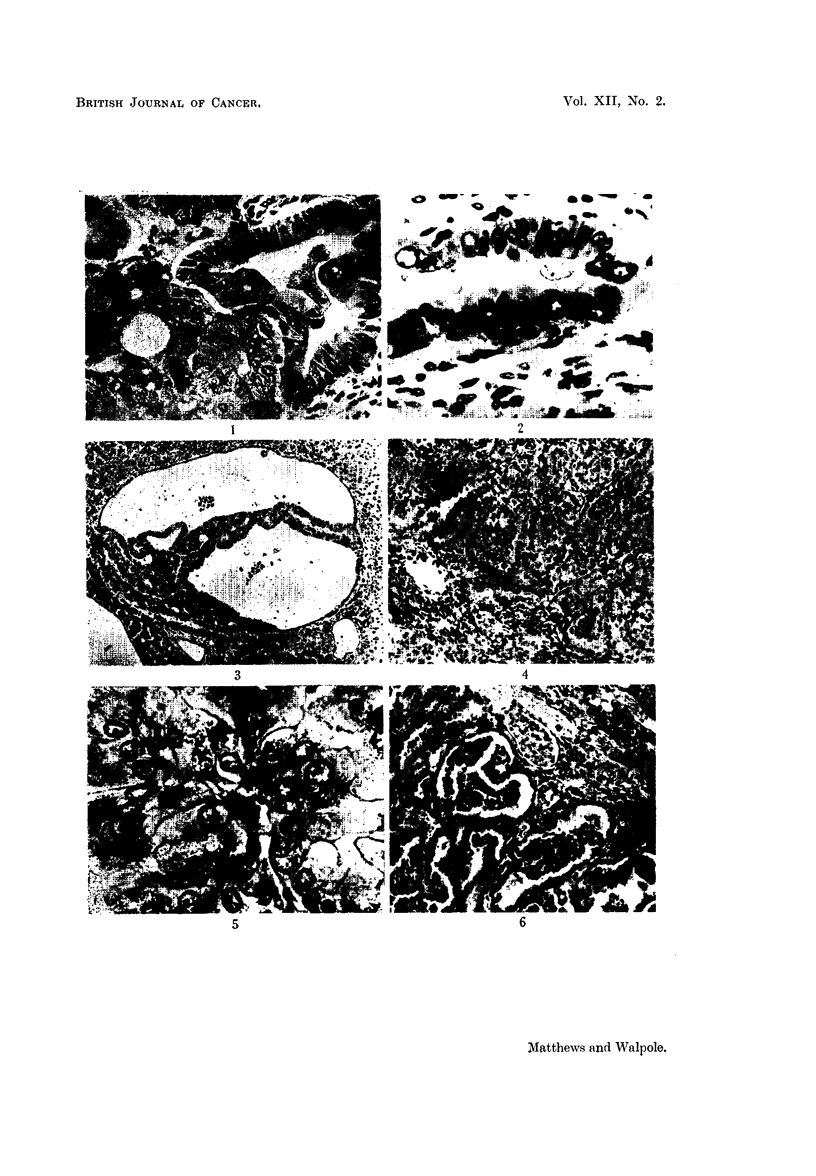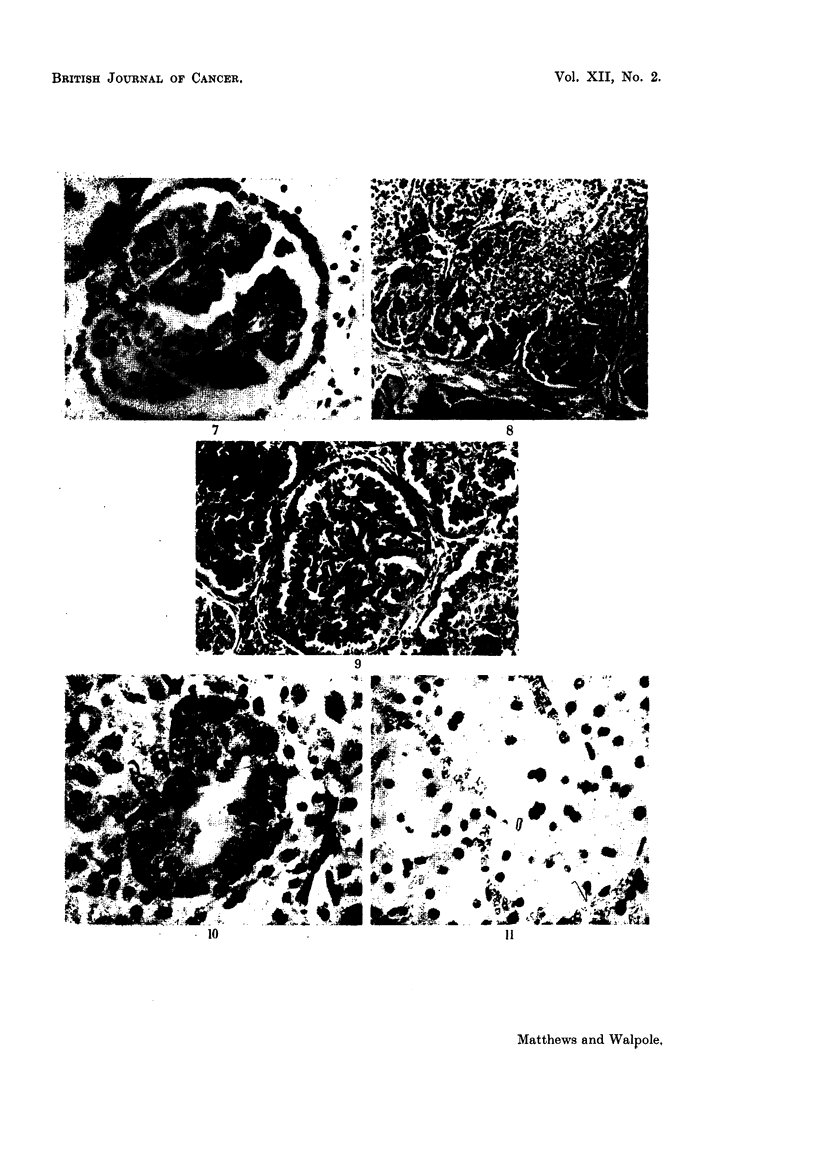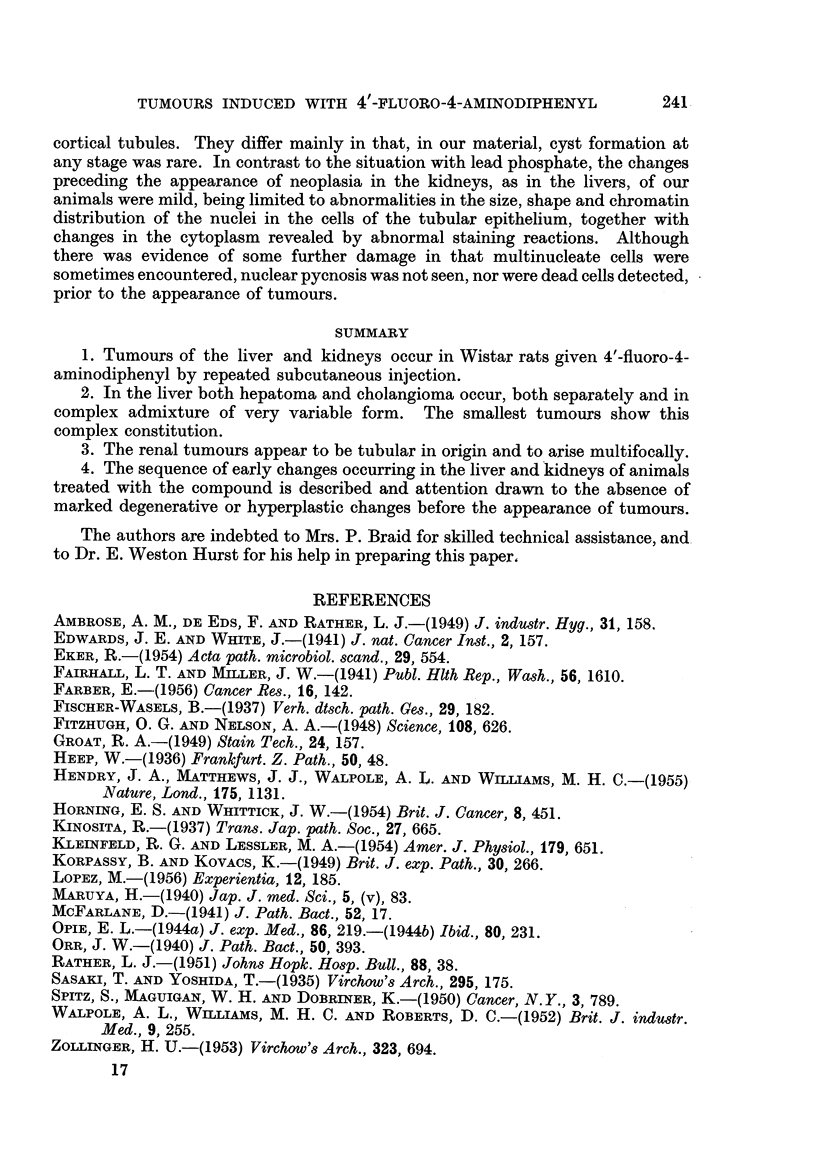# Tumours of the Liver and Kidney Induced in Wistar Rats with 4′-Fluoro-4-Aminodiphenyl

**DOI:** 10.1038/bjc.1958.28

**Published:** 1958-06

**Authors:** Joyce J. Matthews, A. L. Walpole

## Abstract

**Images:**


### TUMOURS OF THE LIVER AND KIDNEY INDUCED IN WISTAR RATS WITH 4′-FLUORO-4-AMINODIPHENYL

JOYCE J. MATTHEWS AND A. L. WALPOLE

*From the Research Department, Imperial Chemical Industries Limited, Pharmaceuticals Division, Alderley Park, Macclesfield, Cheshire*

Received for publication February 11, 1958

IN an attempt to explain the production of intestinal tumours in rats dosed with 4-aminodiphenyl and some of its nuclear-methylated derivatives, Walpole, Williams and Roberts (1952) suggested that the ultimate carcinogen in each instance is the corresponding *ortho*-hydroxy amine, formed in the liver and excreted in the bile. To account for the relatively low yield of tumours in the rat with 4-aminodiphenyl itself, they further suggested that in this species it is largely metabolised to the 4′-hydroxy derivative and that little of the *ortho*-hydroxy amine, 3-hydroxy-4-aminodiphenyl, is formed. These suggestions are represented diagrammatically below, the main metabolic product being indicated by the solid arrow and the carcinogenic metabolite in heavy print.

While metabolic studies were in progress it seemed that some evidence bearing upon these ideas might be had by testing for carcinogenic activity 4′-fluoro-4-aminodiphenyl.

With this substance the possibility of metabolic hydroxylation in the 4′-position is excluded by the presence there of firmly bound fluorine and a relatively high yield of the corresponding *ortho*-hydroxy amine (4′-fluoro-3-hydroxy-4-amino-diphenyl) may be expected. If the suggestions outlined above are correct, this should result in 4′-fluoro-4-aminodiphenyl being more potent as a carcinogen than the parent amine of the series.

The results of our first experiment with this compound have been briefly reported (Hendry, Matthews, Walpole and Williams, 1955). Given to rats in a lower total dose than 4-aminodiphenyl it gives a higher yield of tumours in a shorter time. While the parent amine, however, causes tumours mainly of the intestines, the organs principally affected by the fluoro derivative are the liver and kidneys. The changes seen in the livers of rats treated with 4′-fluoro-4-aminodiphenyl differ in some respects from those usually met with during the induction of liver tumours with chemical agents. Renal tumours, either spontaneous or induced, are uncommon in rats. We have therefore undertaken a closer study of the sequence of changes in the affected organs and to this end a second series of rats was dosed as before and groups killed at various intervals. Our object in this paper is to describe and, as far as possible, to interpret the pathological changes seen in these two series of animals.

### MATERIALS AND METHODS

In both experiments male albino rats were used, of the Wistar strain bred in these laboratories for several years. In the first, 24 rats (mean weight, 117 g.) were given 4′-fluoro-4-aminodiphenyl, dissolved in arachis oil, B.P., by subcutaneous injection in the flank, injections being made daily, Mondays to Fridays inclusive, and continued for about 20 weeks. The initial daily dose of 5 mg. per 100 g. body weight was reduced progressively during the first 7 weeks, on account of toxicity, to 1 mg. per 100 g. per day. One rat died at 48 days; the remainder were killed, as palpable tumours were detected, between 381 and 626 days from the start (total dose, 160 to 240 mg. per 100 g.). Tissues were fixed in acid Zenker and stained with Groat's (1949) haematoxylin and eosin. Liver and kidney from some animals, after appropriate fixation, were stained also with van Gieson's and the periodic acid-Schiff stain and by the Feulgen-aniline blue method of H. W. Altmann.*

In the second experiment 27 rats were used, similar in all respects to those in the first. Eight were given only 3 daily doses of 5 mg. per 100 g. and killed 4 to 6 days after the first dose. The remainder were dosed precisely as before and killed in twos or threes at intervals of 28 days from the start. As tumours had already appeared in both livers and kidneys by the fifth month, all surviving rats were killed at 6 months. The stains used on the tissues from these animals included, in addition to those already mentioned, Masson's trichrome, Best's carmine and toluidine blue, and various procedures specific for iron.

### RESULTS

### *Liver*

Twenty-two of the first group of animals had liver tumours, often multiple and without predominance in any one lobe. The largest were about 4·0 cm. in diameter.

Histologically both hepatomata and cholangiomata are seen, occurring either separately or in admixture, as well as nodular hyperplasia and areas of coarse

* In this method tissues fixed in Zenker-formol are stained by the standard Feulgen technique, mordanted in 5 per cent aqueous phosphotungstic acid, counterstained in acidified aqueous aniline blue with orange G and differentiated in 95 per cent alcohol. The counterstain is made by dissolving 0·5 g. of water soluble aniline blue and 2 g. of gold orange G in 200 ml. of water and adding 8 ml. of acetic acid. It is diluted with 1 to 3 parts of water before use.

collagenous connective tissue containing spaces bounded by a flattened or cubical epithelium. The latter are probably dilated bile-ducts. (Similar areas have been noted in old rats given arachis oil alone and may well be due to age.) In a hyperplastic area in the liver of a single rat there is excessive proliferation of bile-ducts and of fusiform cells which ramify widely between the cords of liver cells.

Usually the tumours in this series are made up of a complex mixture of both hepatomatous and cholangiomatous elements (Fig. 1) and the identity of many of their cells is in doubt. A single cystic space may be lined in part by cells of parenchymal origin and in part by others resembling bile-duct epithelium or by cells whose origin cannot be clearly defined (Fig 2). Occasionally a dilated " bile-duct " contains a plug of hepatoma cells (Fig. 3), or an alveolus of hepatomatous cells a clump of cells of the bile-duct type. Several microscopic tumours display mixed cholangiomatous and hepatomatous alveoli radiating from a central mass even in the initial stages of infiltrating the surrounding liver tissue.

Fibrous stroma is not present in appreciable amount in any of the tumours, least of all the purely hepatomatous areas. Nor is cirrhosis evident in those parts of the liver which have not undergone neoplastic change. Mitoses are for the most part infrequent. Metastases are not seen, although in one section a central vein contains a plug of hepatoma cells. In only one tumour is there any indication of malignancy. This, cytologically, resembles an adenocarcinoma; glycogen is totally absent and mitoses are numerous, but even here no metastases were found.

The livers of the rats from the second experiment were examined carefully in an attempt to trace the histogenesis of the tumours. The earliest change is seen in the nuclei of the liver cells which show much variation in size and shape as early as four days after the start of dosing. Later the number of nuclei affected increases and in many of them the nucleolus is enlarged. After only six days the amount of material stained orange-yellow by Altmann's stain is variable from area to area, as is the amount of tigroid substance. No one part of the liver is particularly affected. These changes persist to the fifth month when the first tumour was observed as a tiny white nodule on the surface of the liver. This was barely a millimetre across, yet proved to be a mixed tumour containing both hepatomatous and cholangiomatous elements. In the remaining animals, killed a month later, numerous small nodules of tumour again showed the complicated appearances described above.

*Kidney*

Kidney tumours were found in 20 of the first series of rats. In 10 they were bilateral and in several instances multiple. Some tumours were immediately visible at autopsy as yellowish or pale grey masses protruding from the surface of the kidneys; others were seen only when the kidneys were sectioned and then appeared as roughly spherical, clearly demarcated structures centred in the cortex. The largest attained a diameter of some 2 cm., the smallest were visible only under the microscope. Since only one kidney section was examined microscopically from each of the rats killed earlier in the experiment, more instances of multiple and bilateral tumours probably occurred than were actually found.

Histologically the tumours seen can be classified into three main types. Those of the first type are composed of swollen, pale-staining cells, with nuclei larger than those of normal tubular cells (Fig. 4 and 5). The nuclei are vesicular and a greater amount of chromatin appears to be concentrated at the nuclear membrane than is usual. The nucleoli also are large. In sections stained with haematoxylin and eosin, the cytoplasm of these cells appears to be less coarsely granular than normal, but not so clear as in the clear-cell type of renal tumour in man. The cells are rarely vacuolated. They are arranged in solid masses and cords, which in places look not unlike adrenal tissue.

Tumours of the second type (Fig. 6 and 7) are composed of cells closely resembling, but rather smaller than those just described. They are arranged in tubules which are often lined by two or more layers of cells. Where only one lining layer is present, the cells are fleshy and a brush-border can be distinguished on a few of them. Individual tubules, particularly at the periphery of these tumours, are dilated and lined by a flattened epithelium from which project papillary ingrowths of smaller, strongly basophilic cells with hyperchromatic nuclei. Scattered amongst these are brightly eosinophilic, necrotic cells. Under low magnification these configurations mimic the appearance of glomeruli.

The third type of tumour (Fig. 8) is composed of small basophilic cells with hyperchromatic nuclei (Fig. 9), arranged in tubules showing a greater tendency to papillary ingrowth. The central portions of these ingrowths are usually necrotic, and necrotic cells are occasionally seen in direct contact with the basement membrane. In one section tumours of all three types are seen lying side by side, separated only by thin bands of compressed kidney tissue.

The centres of the larger tumours of all types are haemorrhagic and frequently contain large blood spaces. Glomeruli are not found within the tumour tissue. Although the tumours are clearly demarcated from the surrounding compressed renal tissue, true encapsulation is not seen. Nevertheless the tumours appear to be benign. Mitotic figures are infrequent, though usually atypical in so far as there is a tendency towards polyploidy, and tripolar spindles occur. " Stickiness " of the chromosomes and bridge formation are not seen. No evidence is found of infiltration or metastatic spread.

All three types of tumour have certain staining properties in common. With haematosylin and eosin the tumour cells appear basophilic, although there is much tinctorial variation. In sections stained with toluidine blue, numerous fine granules are seen in the cytoplasm of tumours of all types. These stain metachromatically and give the cells a reddish-purple colour contrasting with the clear blue-grey of the normal kidney cells. Stained by Altmann's method, tumour cells appear clear blue whereas numerous orange-yellow rods or granules are seen in the basal two-thirds of non-neoplastic tubular cells of the renal cortex.

Sometimes single tubules show the distinctive staining just described; the toluidine blue and the Altmann methods especially distinguish these from the surrounding tissue. One such tubule (Fig. 10), was traced through thirty-two sections. It stood out from the adjacent tissue not only because of its staining properties, but also because its cells were high and fleshy and had large nuclei. Their pattern of chromatin was similar to that seen in the tumours and their nucleoli also were enlarged.

In the smaller tumours in particular coarse yellow-brown cytoplasmic granules are seen. These are not bi-refringent and some give a positive reaction for iron. Granules of this kind are sometimes seen in the convoluted tubules of untreated rats of our stock and are similar in appearance and staining properties to those described in the kidneys of rats treated with certain lead compounds on the one hand (Fairhall and Miller, 1941) and with butter yellow on the other (Kinosita, quoted by Edwards and White, 1941). Eosiniphilic inclusions are occasionally seen in the cytoplasm of the tumour cells, but not within the nucleus as has been reported in animals treated with lead compounds. They stain faintly with P.A.S., but not after pretreatment with diastase; they stain a rosy red with van Gieson and green to purplish-red with Masson. They are especially numerous in areas of necrosis and are similar in staining reactions to the bi-refringent lipids seen by Horning and Whittick (1954) in tumours of the kidney in the golden hamster.

In the second experiment outlined above, we attempted to trace the development of renal tumours in rats given 4′-fluoro-4-aminodiphenyl. The most marked change noted during the first four months is great variation in size, shape and chromatin content of the nuclei of the cells of both cortical and medullary tubules. In addition multinucleate cells appear in some cortical tubules (Fig. 11) either singly or partially lining a tubule. In the third month there is an increase in the intensity of staining of the inner cortical tubules, zone II (McFarlane, 1941) and metachromatic particles are seen in the cytoplasm in sections stained with toluidine blue. In the same areas, and in patches in the outer cortex also, the particulate material stained orange-yellow by the Altmann technique is partially lost. In addition, the yellowish-brown particles already referred to are seen more consistently and in greater amount from the third month onwards. At five months individual cortical tubules rather abruptly assume all the characteristics of the abnormal single tubules already described.

The remaining rats were killed at six months. Small tumours consisting of several tubules lying side by side are seen and with the aid of serial sectioning and block reconstruction we were able to show that these sometimes consist of single tubules and sometimes of several separate tubules. Single tubules are also seen which are dilated and lined by several layers of cells. We have not detected neoplastic changes involving less than a complete tubule. We have ascertained, however, that the change affects both adjacent and widely separated tubules simultaneously and without severe pathological change preceding the appearance of neoplastic changes. In no instance was there destruction of kidney tissue before tumour formation.

*Other organs*

Eight rats in the first series developed intestinal tumours, mostly of the large intestine. These comprised sessile and polypoid adenomata and submucosal granulomata associated with disorganisation and hypertrophy of the glands. They were indistinguishable from neoplasms resulting in the intestines of the rat from the administration of 4-aminodiphenyl and its 3 : 2′-dimethyl homologue (Walpole, Williams and Roberts, 1952). None of the second group of animals showed any changes in the intestines so that no further evidence was obtained of changes preceding those described.

In several animals of the first group microscopic examination revealed adenomata of the pancreatic acinar tissue.

Tumours were also observed at the injection site and in the testes of some of the animals of the first group, killed towards the end of experiment. The former were almost invariably sarcomata of the spindle-cell type, the latter, interstitial cell tumours. The testicular tumours appeared earlier and more frequently than is normal for rats of our stock.

### DISCUSSION

A remarkable feature of these results is the absence of gross pathological change either in the liver or kidneys of rats treated with 4′-fluoro-4-aminodiphenyl before the rather sudden appearance of tumours of these organs.

Tumours of the liver have been induced in rats or mice with a variety of chemical substances and with most of those so far described the appearance of tumours is preceded by well-marked degenerative and hyperplastic reactions within the liver. In drawing attention to the similarity of the early hepatic response in animals treated with many of these substances, Farber (1956) divides the changes seen in common in rats given ethionine, 2-acetamidofluorene, or 3′-methyl-4-dimethylaminoazobenzene into two major sequences. The first involves proliferation of " oval cells ", spreading from the portal areas to involve most of the liver lobule and accompanied by " piecemeal " necrosis of hepatic parenchymal cells, and the second consists of nodular regenerative hyperplasia of liver cells with progressive distortion of the characteristic lobular pattern of the liver.

Similar, if not identical sequences were observed by Kinosita (1937), Maruya (1940) and Orr, (1940) in the livers of rats given 4-dimethylaminoazobenzene (butter yellow), and one or more of the changes described have been seen in the early stages of hepatic carcinogenesis with *o*-aminoazotoluene (Sasaki and Yoshida, 1935; Heep, 1936; Fischer-Wasels, 1937; and Orr, 1940), with benzidine (Spitz, Maguigan and Dobriner, 1950), with tannic acid (Korpassy and Kovacs, 1949) and with thioacetamide (Fitzhugh and Nelson, 1948; Ambrose, de Eds and Rather, 1949; Rather, 1951; Kleinfeld and Lessler, 1954; and Lopez, 1956).

It is evident from these various reports that, in the early reaction of the liver of rats to hepatic carcinogens, several features persistently recur. At the same time widely divergent views have been expressed, first, as to the identity of the cells involved in the early periportal hyperplasia so often described; second, as to the nature of the various hyperplastic changes which occur before neoplasia, i.e. whether or not they are preceded by and resultant upon gross cell damage; and lastly, as to the significance for the process of carcinogenesis of the various tissue-reactions seen. Farber (1956) concludes that neither diffuse nor regional necrosis nor fibrosis is essential to the carcinogenic process and that progressive " oval cell " hyperplasia and the associated atrophic and hypertrophic changes in the adjacent parenchymal liver cells are not by themselves premalignant, but may be necessary in " setting the stage " for the later regenerative and neoplastic response.

Our results with 4′-fluoro-4-aminodiphenyl support the first of these conclusions, inasmuch as neither necrosis nor fibrosis were seen in the livers of rats treated with it before the rather abrupt appearance of hepatic tumours. However, since no early proliferation of " oval cells ", nor indeed of cells of any type, was in evidence in the portal tracts of these animals, this also cannot be regarded as indispensable for an ultimate neoplastic response. The most marked changes noted before the appearance of tumours were in the size, shape and chromatin distribution of the nuclei of hepatic parenchymal cells, together with changes in the cytoplasm revealed by abnormal staining reactions. Nuclear pycnosis was not seen. These changes affected all parts of the liver but were patchy in distribution. The areas in which they occurred could not be correlated with those in which tumours developed, but it seems likely that they represent an essential part of the processes culminating in neoplasia. It has not yet proved possible to define precisely the more subtle modifications in cellular organisation which are reflected in changes of this kind.

The tumours which appeared in the livers of our animals could not be classified into well-defined types as seems to be the case with those produced by carcinogenic azo-compounds (Orr, 1940; Opie, 1944*a*, 1944*b*; Edwards and White, 1941). Mixed tumours containing both hepatomatous and cholangiomatous elements were common, and the fact that even the smallest or earliest tumours might have this complex composition suggests either that the cells stimulated to neoplasia are bi-potential or that the cells of both bile-ducts and parenchyma are simultaneously involved in the neoplastic change.

References to spontaneous renal neoplasms in rats have been collected by Eker (1954), who in addition described a number of familial renal adenomata; mostly multiple and bilateral, in Wistar rats. These tumours occurred as simple cysts, papillary cystadenomata, solid eosinophilic adenomata and solid tubular basophilic adenomata. None contained glomeruli, and Eker suggested that they developed by proliferation of the renal tubular epithelium with or without preliminary cystic dilatation of the same. Rather similar tumours have been reported by Zollinger (1953) as resulting in rats from the repeated subcutaneous injection of lead phosphate—an observation which we have confirmed (Walpole, unpublished). Their appearance, however, was preceded by severe pathological changes in the kidney cortex, in particular the occurrence of numerous cysts, involving mainly the proximal convoluted tubules, and of bizarre nuclear abnormalities in the tubular epithelium. In addition several of the tumours seen by Zollinger were undoubtedly malignant and metastases were found.

Spontaneous renal tumours in rats of our strain are very rare. In the kidneys of several hundred control animals of all ages we have only once or twice encountered primary neoplasms of any type. Those induced with 4′-fluoro-4-aminodiphenyl are benign adenomata. They closely resemble some of those described by Eker and, like those, appear to arise by proliferation of the epithelium of the

---

EXPLANATION OF PLATES

FIG. 1.—Tumour of the liver. Mixed hepatoma (below, left) and duct-like spaces lined by tall columnar epithelium. H. & E. × 265.

FIG. 2.—Tumour of the liver, showing duct-like spaces lined in part by columnar epithelial cells and for the rest by hepatoma cells. H. & E. × 460.

FIG. 3.—Tumour of the liver. A space resembling a dilated bile-duct is being invaded by a mass of hepatoma cells, which can be seen replacing the bile-duct epithelium in several places. H. & E. × 65.

FIG. 4.—Tumour of kidney, type I, showing pale, swollen cells with chromatin concentrated at nuclear membrane and prominent nucleoli. H. & E. ×100.

FIG. 5.—Ditto. H. & E. ×650.

FIG. 6.—Tumour of kidney, type II, showing pseudoglomerular configurations. H. & E. × 120.

FIG. 7.—Ditto. H. & E. × 300.

FIG. 8.—Tumour of kidney, type III, showing papilliform arrangement of cells, which are slightly smaller than those of the uncompressed kidney tissue. H. & E. × 115.

FIG. 9.—Ditto. H. & E. × 157.

FIG. 10.—Section of kidney tubule (or tubules) having the appearance and staining characteristics of some of the larger tumours of the kidney. Appearance due to metachromatic granules. Note also circle of granules to right of tubule. These stain yellow-brown with H. & E. and give a positive reaction for iron. Toluidine blue. × 420.

FIG. 11.—Early change in kidney tubules. Note very much enlarged cells almost blocking tubules, multinucleate cells, and cells with hyperchromatic nuclei. H. & E. × 437.

1

2

3

4

5

6

7

8

9

10

11

Matthews and Walpole.

cortical tubules. They differ mainly in that, in our material, cyst formation at any stage was rare. In contrast to the situation with lead phosphate, the changes preceding the appearance of neoplasia in the kidneys, as in the livers, of our animals were mild, being limited to abnormalities in the size, shape and chromatin distribution of the nuclei in the cells of the tubular epithelium, together with changes in the cytoplasm revealed by abnormal staining reactions. Although there was evidence of some further damage in that multinucleate cells were sometimes encountered, nuclear pycnosis was not seen, nor were dead cells detected, prior to the appearance of tumours.

### SUMMARY

1. Tumours of the liver and kidneys occur in Wistar rats given 4′-fluoro-4-aminodiphenyl by repeated subcutaneous injection.

2. In the liver both hepatoma and cholangioma occur, both separately and in complex admixture of very variable form. The smallest tumours show this complex constitution.

3. The renal tumours appear to be tubular in origin and to arise multifocally.

4. The sequence of early changes occurring in the liver and kidneys of animals treated with the compound is described and attention drawn to the absence of marked degenerative or hyperplastic changes before the appearance of tumours.

The authors are indebted to Mrs. P. Braid for skilled technical assistance, and to Dr. E. Weston Hurst for his help in preparing this paper.

### REFERENCES

Ambrose, A. M., de Eds, F. and Rather, L. J.—(1949) *J. industr. Hyg.*, **31**, 158.
Edwards, J. E. and White, J.—(1941) *J. nat. Cancer Inst.*, **2**, 157.
Eker, R.—(1954) *Acta path. microbiol. scand.*, **29**, 554.
Fairhall, L. T. and Miller, J. W.—(1941) *Publ. Hlth Rep., Wash.*, **56**, 1610.
Farber, E.—(1956) *Cancer Res.*, **16**, 142.
Fischer-Wasels, B.—(1937) *Verh. dtsch. path. Ges.*, **29**, 182.
Fitzhugh, O. G. and Nelson, A. A.—(1948) *Science*, **108**, 626.
Groat, R. A.—(1949) *Stain Tech.*, **24**, 157.
Heep, W.—(1936) *Frankfurt. Z. Path.*, **50**, 48.
Hendry, J. A., Matthews, J. J., Walpole, A. L. and Williams, M. H. C.—(1955) *Nature, Lond.*, **175**, 1131.
Horning, E. S. and Whittick, J. W.—(1954) *Brit. J. Cancer*, **8**, 451.
Kinosita, R.—(1937) *Trans. Jap. path. Soc.*, **27**, 665.
Kleinfeld, R. G. and Lessler, M. A.—(1954) *Amer. J. Physiol.*, **179**, 651.
Korpassy, B. and Kovacs, K.—(1949) *Brit. J. exp. Path.*, **30**, 266.
Lopez, M.—(1956) *Experientia*, **12**, 185.
Maruya, H.—(1940) *Jap. J. med. Sci.*, **5**, (v), 83.
McFarlane, D.—(1941) *J. Path. Bact.*, **52**, 17.
Opie, E. L.—(1944*a*) *J. exp. Med.*, **86**, 219.—(1944*b*) *Ibid.*, **80**, 231.
Orr, J. W.—(1940) *J. Path. Bact.*, **50**, 393.
Rather, L. J.—(1951) *Johns Hopk. Hosp. Bull.*, **88**, 38.
Sasaki, T. and Yoshida, T.—(1935) *Virchow's Arch.*, **295**, 175.
Spitz, S., Maguigan, W. H. and Dobriner, K.—(1950) *Cancer, N.Y.*, **3**, 789.
Walpole, A. L., Williams, M. H. C. and Roberts, D. C.—(1952) *Brit. J. industr. Med.*, **9**, 255.
Zollinger, H. U.—(1953) *Virchow's Arch.*, **323**, 694.